# Pretreatment with Fish Oil-Based Lipid Emulsion Modulates Muscle Leukocyte Chemotaxis in Murine Model of Sublethal Lower Limb Ischemia

**DOI:** 10.1155/2017/4929346

**Published:** 2017-01-15

**Authors:** Yao-Ming Shih, Juey-Ming Shih, Yu-Chen Hou, Chiu-Li Yeh, Cheng-Che Li, Sung-Ling Yeh

**Affiliations:** ^1^School of Nutrition and Health Sciences, College of Nutrition, Taipei Medical University, Taipei, Taiwan; ^2^Department of Surgery, Cathay General Hospital, Taipei, Taiwan; ^3^Master Program in Food Safety, College of Nutrition, Taipei Medical University, Taipei, Taiwan; ^4^Department of Nutrition and Health Sciences, Chinese Culture University, Taipei, Taiwan; ^5^Nutrition Research Center, Taipei Medical University Hospital, Taipei, Taiwan

## Abstract

This study investigated the effects of a fish oil- (FO-) based lipid emulsion on muscle leukocyte chemotaxis and inflammatory responses in a murine model of limb ischemia-reperfusion (IR) injury. Mice were assigned randomly to 1 sham (sham) group, 2 ischemic groups, and 2 IR groups. The sham group did not undergo the ischemic procedure. The mice assigned to the ischemic or IR groups were pretreated intraperitoneally with either saline or FO-based lipid emulsion for 3 consecutive days. The IR procedure was induced by applying a 4.5 oz orthodontic rubber band to the left thigh above the greater trochanter for 120 min and then cutting the band to allow reperfusion. The ischemic groups were sacrificed immediately while the IR groups were sacrificed 24 h after reperfusion. Blood, IR-injured gastrocnemius, and lung tissues were collected for analysis. The results showed that FO pretreatment suppressed the local and systemic expression of several IR-induced proinflammatory mediators. Also, the FO-pretreated group had lower blood Ly6C^hi^CCR2^hi^ monocyte percentage and muscle M1/M2 ratio than the saline group at 24 h after reperfusion. These findings suggest that FO pretreatment may have a protective role in limb IR injury by modulating the expression of proinflammatory mediators and regulating the polarization of macrophage.

## 1. Introduction

Acute limb ischemia is a frequently encountered complication following peripheral arterial embolism, vascular trauma, or intraoperative tourniquet use [1]. Reestablishment of blood flow to the ischemic tissue is important to restore tissue viability but the process of reperfusing the ischemic tissue carries the risk of inducing local tissue and remote organ injury [2]. The mechanisms of ischemic reperfusion (IR) injury are multifactorial in nature and experimental treatment strategies have been designed to ameliorate components of IR injury including complement activation [3], neutrophil infiltration [4], reactive oxygen species [5], metabolic failure [6], and inflammation [7].

It has been demonstrated that the severity of muscle damage after IR injury is correlated directly with the number of leukocyte infiltration [8, 9] which involves cytokine production, upregulation of adhesion molecules, and chemokine expression [10]. Neutrophils in blood express high levels of chemokine receptor CXCR2. Rapid neutrophil mobilization to sites of inflammation is mediated by CXCR2 ligands such as keratinocyte-derived chemokine (KC) and macrophage inflammatory protein 2 (MIP-2) in mouse and growth-related oncogene *α* in human [11]. Recent studies have focused on the importance of monocyte differentiation and infiltration into injured tissue and the significance of the macrophage polarization state in tissue repair after ischemic stroke [9, 10]. Monocytes/macrophages are heterogeneous cell populations. Classically, the myeloid-derived monocytes can be divided into two subsets. The “inflammatory” Ly6C^hi^CCR2^hi^ (Ly6C^+^) monocytes known as the classical subset can extravasate into inflamed tissues and differentiate into inflammatory macrophages, while the Ly6C^low^CCR2^low^ (Ly6C^−^) monocytes or the “patrolling” monocytes function as surveillance immune cells and have been suggested to contribute to the repair of vascular endothelial damage [12]. During inflammation, blood Ly6C^+^ monocytes carrying chemokine receptor CCR2 are recruited to injured tissues that express high level of monocyte chemoattractant protein-1 (MCP-1/CCL2). These recruited monocytes serve as a source of inflammatory macrophages and are thought to exert a proinflammatory role in acute tissue injury [9]. An important function of the macrophages is to maintain tissue integrity, which is modulated by the surrounding cytokine microenvironment that determines M1 and M2 macrophage differentiation. The M1 (classically activated proinflammatory) macrophages promote inflammation and cause tissue damage, while the M2 (alternatively activated anti-inflammatory) macrophages encourage cell proliferation and tissue repair [13]. Studies have demonstrated that the processes of adequate macrophage recruitment followed by appropriate polarization switch to M2 subtype are important processes for skeletal muscle repair [14, 15].

Fish oils (FO) are rich sources of eicosapentaenoic acid (EPA) and docosahexaenoic acid (DHA). These long-chain n-3 polyunsaturated fatty acids (PUFAs) were shown to possess immunomodulatory and anti-inflammatory properties [16]. Previous studies investigating the effects of FO on limb IR injury are scarce. However, possible benefits of n-3 PUFAs have been demonstrated in a human cohort study of patients with peripheral artery disease (PAD). In that study, n-3 PUFA content in red blood cells was found to be inversely associated with plasma level of inflammatory biomarkers [17]. Recently, a study by Hishikari et al. demonstrated that lower serum EPA/arachidonic acid ratio was associated with a greater risk of major adverse limb events in patients with PAD [18]. In animal studies assessing intestinal IR injury, supplementation of FO-containing lipid emulsions has also been demonstrated to exhibit protective effects by modulating inflammation and macrophage infiltration [19]. Our study is the first to assess the effects of a FO-based lipid emulsion on local skeletal muscle and remote lung injury after reperfusion in a murine model of unilateral limb ischemia. A special emphasis of this investigation was to understand the recruitment pattern of various leukocyte subpopulations and the status of macrophage polarization switch during the period after reperfusion. We hypothesized that FO may ameliorate local skeletal tissue damage, reduce systemic inflammatory response, and protect against remote lung injury from limb IR by modulating cytokine/chemokine expressions and altering leukocyte activation. This issue may be of interest to patients recovering from recent acute limb ischemia.

## 2. Materials and Methods

### 2.1. Animals

Forty male C57BL/6 mice (5 weeks old, weighing 18–20 g) were obtained from the National Laboratory Animal Center (Taipei, Taiwan). Before use in the study, animals underwent an acclimatization period for 4 weeks. All mice were housed in a temperature- and humidity-controlled room and maintained on a 12 h light-dark cycle with food and water available ad libitum. Care of laboratory animals was in full compliance with the Guide for the Care and Use of Laboratory Animals (National Research Council, 1996). Experiment protocols were approved by the institution's Animal Care and Use Committee at Taipei Medical University.

### 2.2. Experimental Design

Mice were assigned randomly to 1 sham group, 2 ischemic groups, and 2 ischemia-reperfusion (IR) groups. The sham group did not undergo the ischemic procedure. Prior to IR procedure, the mice in the ischemic or IR groups were administered intraperitoneally (IP) with either saline or FO-based lipid emulsion at 10 mL/kg body weight/day (10% Omegaven, Fresenius-Kabi, Homburg, Germany) for 3 consecutive days. FO-based lipid emulsion was provided as a supplement in addition to standard chow feed ad libitum (Purina number 5001). The fatty acid composition of the FO-based lipid emulsion contains C14:0, 4.9%; C16:0, 10.7%; C18:0, 2.4%; C16:1 n-7, 8.2%; C18:1 n-9, 12.3%; C18:2 n-6, 3.7%; C18:3 n-3, 1.8%; C20:4 n-6, 2.6%; C20:5 n-3, 18.8%; C22:5 n-3, 2.8%; C22:6 n-3, 16.5%; others, 15.3% according to the manufacturer. The dose of FO used is equivalent to 1 g/kg BW/day or 0.3 g/kg/day in humans [20], a dosage demonstrated to be therapeutically effective in humans [21]. The animals were subjected to hind limb ischemia for 120 min followed by 24 h reperfusion. The ischemic groups were sacrificed immediately at the start of reperfusion (0 h), while mice in the IR groups were sacrificed at 24 h after reperfusion. Standard chow diet and daily IP injection of either saline or FO-based lipid emulsion were administered during the experimental period (3 days prior to induction of ischemia). There were 5 groups in this study: sham group (sham), mice that underwent shaving but without banding; S0 group (S0), ischemic mice pretreated with saline and sacrificed immediately after onset of reperfusion; F0 group (F0), ischemic mice pretreated with FO-based lipid emulsion sacrificed immediately after onset of reperfusion; S24 group (S24), ischemic mice pretreated with saline sacrificed at 24 h after reperfusion; and finally F24 group (F24), ischemic mice pretreated with FO-based lipid emulsion sacrificed at 24 h after reperfusion. There were 8 mice in each respective group.

### 2.3. IR Procedure

The induction of ischemia performed was a procedure modified based on the model described by Crawford et al. [22]. Briefly, animals were deeply anesthetized with an IP injection of Zoletil (25 mg/kg) and Rumpun (10 mg/kg), followed by the application of a 4.5 oz orthodontic rubber band (ORB; American Orthodontics, Sheboygan, WI) to the left thigh above the greater trochanter. All mice received 0.5 mL saline subcutaneously for hydration upon initial banding. During the ischemic period, animals were returned back to their respective cages in the animal housing facility. Mice were maintained in a lateral decubitus position and kept anesthetized throughout the course of the ischemic period. After 120 min of ischemia, reperfusion was initiated by cutting the rubber band with scissors and the animals were allowed to recover from anesthesia. An additional 0.5 mL was given subcutaneously at the beginning of the reperfusion period in all IR groups. Mice in the sham group underwent anesthesia and shaving of the left hind limb but did not undergo the IR procedure. At the end of the respective experimental periods, mice were euthanized by cardiac puncture. Blood samples were collected in tubes containing heparin. Muscle (gastrocnemius) tissues of the affected limb were obtained for subsequent biochemical analysis. The removed lung tissues were snap frozen in liquid nitrogen and stored at −80°C for further analysis.

### 2.4. Plasma Chemokines and Cytokines

Components of whole blood were separated by centrifugation at 1500 ×g at 4°C for 10 min to obtain the plasma. Measurements of monocyte chemoattractant protein- (MCP-) 1, macrophage inflammatory protein- (MIP-) 2, and interleukin- (IL-) 6 concentrations were performed using a commercially available enzyme-linked immunosorbent assay (ELISA) kit (ICL, Newberg, OR). Described briefly, antibodies (Abs) specific to mouse MCP-1, MIP-2, and IL-6 were first coated onto the wells of the microtiter strips, which were then incubated with plasma samples. The antibody-bound samples were later developed with reagents, with the absorbance of each well read using a spectrophotometer.

### 2.5. Distribution of Blood Leukocytes and Their Expression of Adhesion Molecules and Chemokine Receptors

Whole blood was used to analyze the distribution of peripheral blood leukocyte subpopulations by flow cytometry. Each blood sample was aliquoted into 2 vials of 100 *μ*L each. One vial was incubated with the following antibodies for analysis of monocytes: PerCP-conjugated anti-CD45 (BioLegend, San Diego, CA, USA), PE-conjugated anti-F4/80 (BioLegend), Pacific blue-conjugated anti-Ly6C (BioLegend), and APC-conjugated anti-CCR2 (R & D Systems, Inc., Minneapolis, MN, USA). The other was incubated with PerCP-conjugated anti-CD45, FITC-conjugated anti-Ly6G, and APC-conjugated anti-CXCR2 (all from BioLegend) for analysis of neutrophils. The concentrations of antibodies chosen were compliant with those recommended by the manufacturer. After incubating the blood sample with the antibodies in the dark at 4°C for 30 min, red blood cells were lysed and separated from the sample. The isolated antibody-stained cells were then resuspended in staining buffer (PBS with 0.5% bovine serum albumin) for analysis, performed with a FACS Canto II flow cytometer (BD Biosciences). CD45 positive cells were gated and interpreted as leukocytes. Leukocyte populations were presented as the percentages of monocytes/macrophages (F4/80^+^) and neutrophils (Ly6G^+^CXCR2^+^) among leukocytes. For analysis of monocyte subpopulations, CD45^+^ F4/80^+^ cells were gated and their subsets were determined by the levels of Ly6C and CCR2 expressed.

### 2.6. Expression of Skeletal Tissue Chemokine and Cytokine Genes Analyzed by Quantitative Real-Time Reverse Transcription Polymerase Chain Reaction

To assess the degree of local skeletal muscle inflammation, we analyzed (1) inflammatory related genes (KC, MCP-1, tumor necrosis factor- (TNF-) *α*, and IL-6) and (2) anti-inflammatory genes (heat shock protein 72 (HSP72) and peroxisome proliferator-activated receptor gamma (PPAR-*γ*)). Glyceraldehyde-3-phosphate dehydrogenase (GAPDH) was used as the housekeeping gene. Total RNA was isolated from muscle tissue using TRIzol reagent (Invitrogen, Carlsbad, CA) followed by chloroform phase separation and 2-propanol precipitation. Isolated RNA (1 *μ*g) was reverse transcribed using oligo (dT) 18 primers with a complementary DNA synthesis kit (Fermentas, Glen Burnie, MD) according to standard protocols. Analysis of real-time polymerase chain reaction (PCR) was carried out using SYBR Green reagents on an ABI 7300 Real-Time PCR system (Applied Biosystems, Foster City, CA). Mouse-specific primer pairs designed for the targeted template are listed in Table 1. The expression of each gene was assayed with the addition of all the following reagents to a final volume of 25 *μ*L: 12.5 uL of 2x Maxima SYBR Green qPCR Master Mix (Fermentas), 200 nM primer, and 100 ng complementary DNA (cDNA). The reaction was carried out by starting with 1 cycle of 2 min at 50°C and 10 min at 95°C followed by 40 cycles of 15 sec at 95°C and 1 min at 60°C, with the process verified by a final dissociation curve (DC) analysis. Cycle threshold (CT) values for each gene of interest were normalized to mice GAPDH and expressions of the messenger (m)RNAs were calculated by the equation 2^−ΔΔCT^.

### 2.7. Distribution of Skeletal Tissue Leukocytes and Expression of Their Adhesion Molecules and Chemokine Receptors

Muscle tissues were digested first in DMEM solution containing 0.1% type II collagenase for 1 h at 37°C. The undigested tissues were then disrupted and filtered through a 70-*μ*m nylon filter (Becton Dickinson, Franklin Lakes, NJ, USA) using a plunger. Isolated cells were resuspended in staining buffer (PBS with 0.5% bovine serum albumin) and incubated with different antibodies to detect leukocytes as described below. The antibodies utilized were as follows: PerCP-conjugated anti-CD45 for leukocytes, PE-conjugated anti-F4/80 for monocytes/macrophages, FITC-conjugated anti-Ly6G for neutrophils, and APC-conjugated anti-CD206 for macrophage subpopulations (all from BioLegend). Stained cells were analyzed with a FACS Canto II flow cytometer (BD Biosciences). Gated CD45-positive cells were considered to be leukocytes. Leukocyte subpopulations were presented as the percentages of macrophages (F4/80^+^) and neutrophils (Ly6G^+^) among leukocytes. The percentages of M1 (CD206^−^) and M2 macrophages (CD206^+^) among the macrophages were determined and the ratio of M1 to M2 macrophages was calculated.

### 2.8. Extraction of Lung Tissue RNA and Analysis by Real-Time Polymerase Chain Reaction (PCR) in Lung

Total RNA was isolated from a 20 mg lung specimen using the TRIzol reagent (Invitrogen, Carlsbad, CA, USA). Complementary (c)DNA was synthesized and amplification was carried out as previously described (in the skeletal muscle tissue assessment section). Primer sequences used for the quantitative RT-PCR assays are listed in Table 1 to include the following: MCP-1, KC, IL-6, and PPAR-*γ*.

### 2.9. Statistical Analysis

All data are expressed as the mean ± standard error of the mean (SEM). All analyses were conducted using GraphPad Prism 5 (GraphPad Software, La Jolla, CA, USA). One-way analysis of variance (ANOVA) with Tukey's post hoc test was used to investigate the differences among groups. A *p* value of <0.05 was considered statistically significant.

## 3. Results 

### 3.1. Inflammatory Mediators in Plasma

MIP-2 in both saline and FO-pretreated groups was elevated severalfold immediately after 120 min of ischemia but returned to baseline levels comparable to the sham group after 24 h of reperfusion. MCP-1 and IL-6 levels, on the other hand, increased significantly only at the end of reperfusion. Furthermore, the increase of MCP-1 and IL-6 levels in the saline-pretreated group was about 2x greater than that of the FO-pretreated group (Table 2).

### 3.2. Blood Leukocyte Distributions

**Figure 1:**
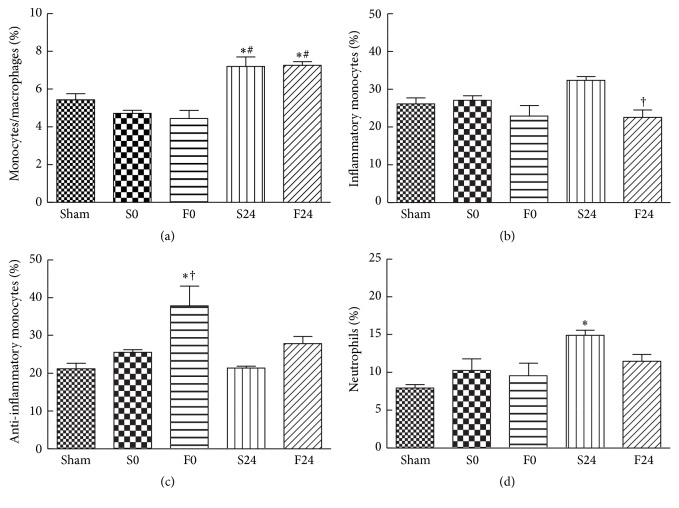
Distribution of blood (a) monocytes/macrophages (F4/80 in CD45), (b) inflammatory monocytes (Ly6C^hi^CCR2^hi^ in CD45 F4/80), (c) anti-inflammatory monocytes (Ly6C^low^CCR2^low^ in CD45 F4/80), and (d) neutrophils (Ly6G^hi^CXCR2^+^ in CD45). For analyzing monocytes/macrophages in blood, mononuclear cells were first identified on the basis of low FSC and SSC. CD45 mononuclear cells were gated and cells expressing F4/80 were further analyzed. Monocyte subpopulations were presented as percentages of Ly6C^hi^CCR2^hi^ and Ly6C^low^CCR2^low^ among CD45 F4/80 mononuclear cells. Population of neutrophils is presented as percentage of Ly6G^hi^CXCR2^+^ in CD45^+^ cells. Sham group, without IR procedure; S0 group, ischemic mice pretreated with saline injection sacrificed immediately after onset of reperfusion (time 0); F0 group, ischemic mice pretreated with FO-based lipid emulsion sacrificed at time 0; S24, ischemic mice pretreated with saline injection sacrificed at 24 h after reperfusion (time 24 h); F24, ischemic mice pretreated with FO-based lipid emulsion sacrificed at time 24 h. Data are presented as the mean ± SEM (*n* = 8). Differences among groups were analyzed by one-way ANOVA with Tukey's post hoc test. ^*∗*^Different from the sham group. ^†^Different from the saline (S) group at the same time point. ^#^Different from other time points in the same group (*p* < 0.05).

Ischemia did not result in elevation of blood monocytes (represented as F4/80 in CD45^+^ cells). The percentage of monocytes in blood increased at 24 h after reperfusion regardless of whether the mice were pretreated with saline or FO (Figure 1(a)). Yet among the increased monocytes, the percentage of inflammatory subtype (represented by Ly6C^hi^CCR2^hi^, Ly6C^+^) in the FO-pretreated group was significantly lower than that of the saline group at the end of reperfusion (Figure 1(b)). In addition, the percentage of anti-inflammatory monocytes (represented by Ly6C^low^CCR2^low^, Ly6C^−^) in the FO-pretreated group showed significant elevation at the end of the ischemic phase (Figure 1(c)). Activated neutrophils expressing the CCR2 receptor (represented by Ly6G^hi^CXCR2^+^), a subpopulation of neutrophils that migrate directly towards the site of inflammatory tissues, showed a significant increase in the saline-pretreated group as compared to sham mice at the end of reperfusion. Such increase, however, was not witnessed in the FO-pretreated group (Figure 1(d)).

### 3.3. Inflammatory Mediators in Muscle

**Figure 2:**
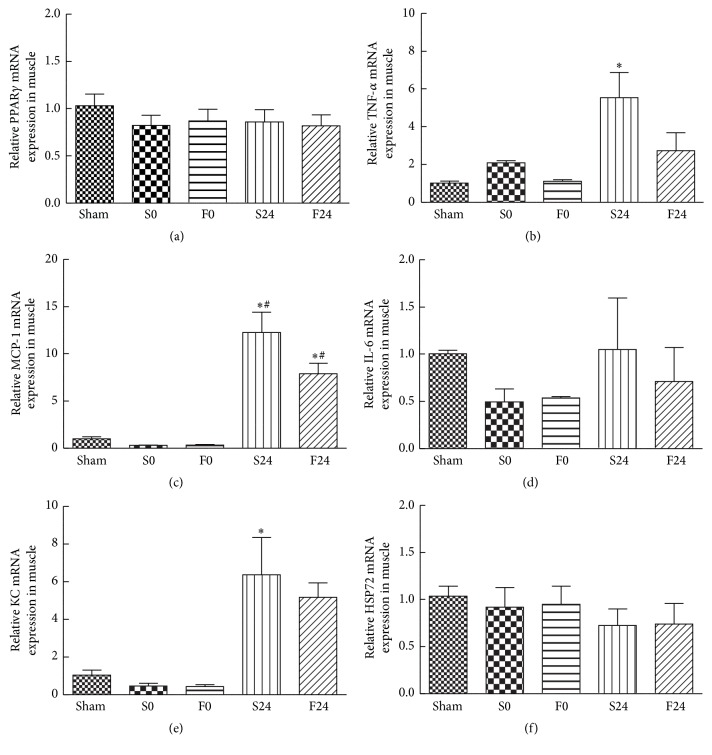
Expression of (a) peroxisome proliferator-activated receptor-*γ* (PPAR-*γ*), (b) tumor necrosis factor-*α* (TNF-*α*), (c) monocyte chemotactic protein-1 (MCP-1), (d) interleukin-6 (IL-6), (e) keratinocyte-derived chemokine (KC), and (f) heat shock protein 72 (Hsp72) mRNAs in muscle tissues. The groups are described in the legend of Figure 1. Data are presented as the mean ± SEM (*n* = 8). Differences among groups were analyzed by one-way ANOVA with Tukey's post hoc test. ^*∗*^Different from the sham group. ^#^Different from other time points in the same group (*p* < 0.05).

The mRNA expression of anti-inflammatory mediators PPAR-*γ* and HSP72 showed no differences between the sham, saline-pretreated, and FO-pretreated groups (Figures 2(a) and 2(f)). In contrast, the expression of proinflammatory mediators was significantly upregulated after 24 h of reperfusion. Compared to the sham group, the saline-pretreated group showed a significant increase in expression of TNF-*α*, MCP-1, and KC mRNAs after 24 h of reperfusion (Figures 2(b), 2(c), and 2(e)). However, in the FO-pretreated groups, the expression of TNF-*α* and KC mRNAs did not differ from the sham group (Figures 2(b) and 2(e)). In contrast to these proinflammatory mediators, induction of IL-6 mRNA expression was not observed in either saline or FO-pretreated groups at both time points (Figure 2(d)).

### 3.4. Leukocyte Distribution in Muscle

**Figure 3:**
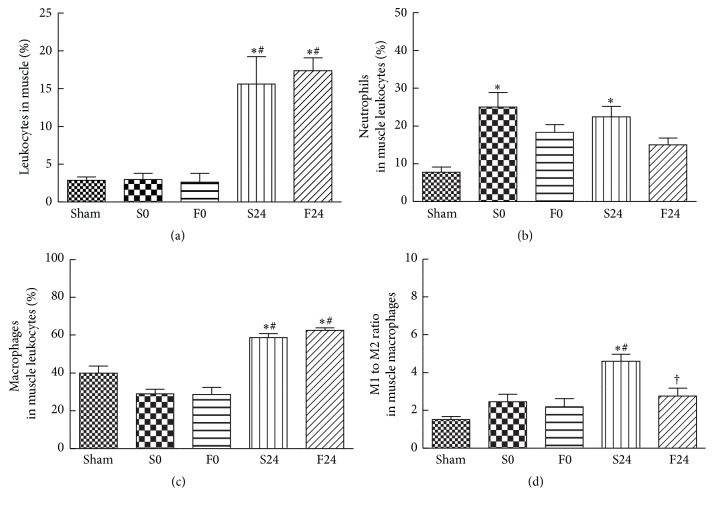
Distribution of (a) white blood cells, (b) neutrophils, (c) macrophages, and (d) M1 to M2 ratio in muscle. Population of neutrophils is presented as percentage of Ly6G in muscle CD45^+^ cells. Population of macrophages is presented as percentage of F4/80 in CD45^+^ cells. M1/M2 ratio is calculated based on the percentages of M1 and M2 in muscle CD45 F4/80. The groups are described in the legend of Figure 1. Data are presented as the mean ± SEM (*n* = 8). Differences among groups were analyzed by one-way ANOVA with Tukey's post hoc test. ^*∗*^Different from the sham group. ^†^Different from the saline (S) group at the same time point. ^#^Different from other time points in the same group (*p* < 0.05).

Evidence of significant leukocyte, neutrophil, and macrophage infiltrations in muscle was noted at 24 h after reperfusion in the saline-pretreated group (Figures 3(a), 3(b), and 3(c)). Interestingly, a significant increase in muscle neutrophil infiltration (represented by Ly6G^+^ CD45 cells) was observed early at the start of reperfusion (Figure 3(b)). At 24 h after reperfusion, there were no differences in leukocyte and macrophage infiltrations between the FO- and saline-pretreated groups. However, neutrophil infiltration in the FO-pretreated groups remained comparable to sham, whereas the saline-pretreated groups showed significant elevations at 0 and 24 h after reperfusion (Figures 3(a), 3(b), and 3(c)). Also, the M1/M2 ratio was significantly lower in the FO-pretreated group after reperfusion than the saline-pretreated group (Figure 3(d)).

### 3.5. Inflammatory Mediators in Lung

**Figure 4:**
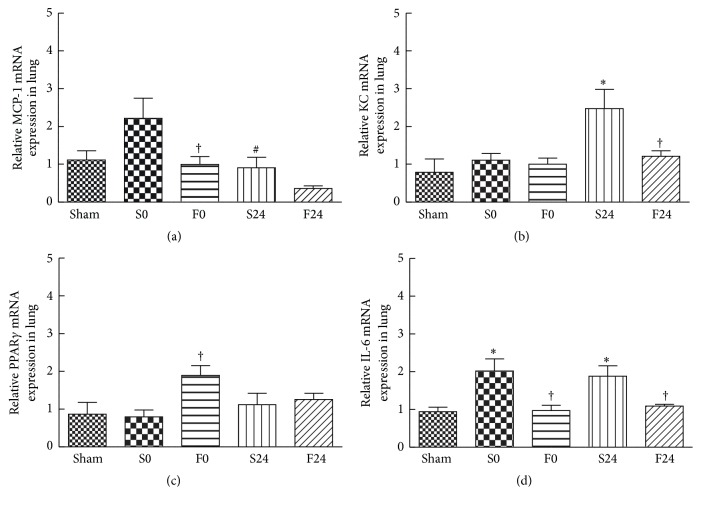
Expression of (a) monocyte chemotactic protein-1 (MCP-1), (b) keratinocyte-derived chemokine (KC), (c) peroxisome proliferator-activated receptor-*γ* (PPAR-*γ*), and (d) interleukin-6 (IL-6) mRNAs in lung tissues. Data are presented as the mean ± SEM (*n* = 8). Differences among groups were analyzed by one-way ANOVA with Tukey's post hoc test. ^*∗*^Different from the sham group. ^†^Different from the saline (S) group at the same time point. ^#^Different from other time points in the same group (*p* < 0.05).

Unlike the late expression seen in muscle tissue, the expression of MCP-1 mRNA in lung tissue of saline-pretreated mice occurred early at the end of ischemia resulting in a 2-fold increase as compared to the sham controls (Figure 4(a)). In addition to MCP-1, IL-6 mRNA expression in the saline-pretreated group also increased significantly after ischemia and this elevation persisted along with a significant increase in the neutrophil chemoattractant, KC (CXCL1) seen at 24 h reperfusion (Figures 4(b) and 4(d)). In contrast, the FO-pretreated groups showed no elevation in MCP-1, KC, and IL-6 mRNA expressions at both time points. However, a significant rise in PPAR-*γ* mRNA was observed immediately after ischemia in the FO-pretreated group (Figure 4(c)).

## 4. Discussion

In this study, we pretreated the mice with FO for 3 days prior to IR injury. This model attempts to simulate the clinical scenario of intraoperative tourniquet use in patients undergoing reconstructive or vascular surgery. We evaluated the outcomes at 24 h after reperfusion because our previous study showed significant local and systemic inflammation and leukocyte activation at this time point [23]. In this study, we found that supplementation with FO-based lipid emulsion few days prior to ischemia-reperfusion had some favorable effects on reducing inflammatory response and modulating macrophage polarization in response to IR injury.

Severity of muscle damage after IR injury can be reflected by the degree of leukocyte infiltration [8, 9] which involves cytokine production, upregulation of adhesion molecules, and chemokine expression [10]. Similar cytokine responses have been demonstrated in critical illness involving sepsis and trauma injury [24]. In this study, mice subjected to 120 min of sublethal ischemia followed by 24 h of reperfusion showed a significant rise in level of proinflammatory cytokine (IL-6) and chemokines (MCP-1 and MIP-2) in plasma (Table 2) and in expression of proinflammatory mRNAs (TNF-*α*, KC, and MCP-1) in muscle tissue (Figures 2(b), 2(c), and 2(e)). In addition, these mice showed significant elevation in the percentage of monocytes/macrophages (F4/80^+^, CD45 cells) and neutrophils (Ly6G^hi^CXCR2^+^, CD45 cells) in blood (Figures 1(a) and 1(d)); exhibited an upregulation of total leukocytes, neutrophils, and macrophages in muscle (Figures 3(a), 3(b), and 3(c)); and demonstrated high M1/M2 ratio in muscle (Figure 3(d)). These findings demonstrated that the presence of prominent systemic and local inflammation was associated with extensive leukocyte infiltration at the inflamed muscle tissues. Furthermore, since remote lung injury had been shown following hind limb IR [25], expression of MCP-1, KC, and IL-6 mRNAs in lung tissues was assessed in this study. The early presence of upregulated inflammatory mediators in lungs demonstrated that inflammation occurred early in remote organs even in the absence of reperfusion. These findings are consistent with our previous histological findings of IR-induced lung injury at 0 h of reperfusion, which included the following: destruction of alveolar structures, neutrophil infiltration, thickening of septal space, and intra-alveolar hemorrhage and debris [23].

There were several beneficial effects in animals pretreated with FO in IR injury. First, FO pretreatment was associated with a significant decrease in plasma MCP-1 and IL-6 after 24 h of reperfusion injury as compared to the saline-pretreated group (Table 2). These findings are consistent with previous studies demonstrating anti-inflammatory benefits of FO under conditions of IR injury [19, 26]. Second, although FO pretreatment did not alter the proportion of monocytes/macrophages in blood (F4/80^+^, CD45 cells), the percentage of “inflammatory” monocyte subpopulation (Ly6C^+^, Figure 1(b)) was reduced at 24 h after reperfusion, whereas the “patrolling” (or anti-inflammatory) monocyte (Ly6C^−^, Figure 1(c)) subpopulation was shown to increase early at the end of ischemia. Ly6C^+^ species were thought to be destructive during the acute inflammatory phase [9], while Ly6C^−^ species have distinctive roles in the resolution of inflammation in the vascular endothelium [12]. Furthermore, FO pretreatment also suppressed the elevation of activated neutrophils in blood (Ly6G^+^, Figure 1(d)). Third, although total leukocyte infiltration in post-IR-injured muscle increased significantly in both the saline- and FO-pretreated groups (Figure 3(a)), Ly6G^+^ neutrophil infiltration in the FO-pretreated group remained comparable to that of the sham controls whereas this phenomenon was not observed in saline-pretreated groups (Figure 3(b)). Moreover, despite concomitant increase in tissue macrophages in both saline- and FO-pretreated groups (Figure 3(c)), the latter showed a significant decrease in the M1/M2 ratio as compared to the saline group indicating the presence of more M2 polarization in muscle tissues after FO pretreatment (Figure 3(d)). The importance of this shift in the macrophage subpopulation to predominately M2 subtype during tissue repair has been implicated recently. In a murine model of ischemic stroke, promotion of M2 macrophages was associated with limiting the extent of brain injury and functional deficit after ischemic stroke [27]. Consistent with the polarization towards M2, muscle inflammation after IR injury was ameliorated in the FO-pretreated groups as demonstrated thru the suppression of KC and TNF-*α* mRNA expressed in muscle. Finally, in contrast to the saline-pretreated group, increases in expression of proinflammatory chemokines and cytokines (MCP-1, KC, and IL-6) in lung tissues were not witnessed in the FO-pretreated group (Figures 4(a), 4(b), and 4(d)). Furthermore, expression of lung PPAR-*γ* mRNA in the FO group was significantly elevated early at the end of ischemic phase (Figure 4(c)). These findings suggest that the early inflammatory process seen in the remote lung tissue of the saline group may be attenuated via the PPAR pathway prior to reperfusion when pretreated with FO.

The effects of FO supplementation responsible for attenuating inflammatory reaction and modulating polarization switch of macrophage may involve several mechanisms. Previous study showed that n-3 PUFA-derived EPA and DHA may ameliorate the severity of acute lung injury by suppressing the production of 4-series leukotrienes and 2-series prostanoids from n-6 PUFAs [28]. Resolvins E1 and D1, the downstream derivatives of EPA and DHA, respectively, have also been shown to possess anti-inflammatory activities that may be protective against bacterial pneumonia and acute lung injury [29, 30]. In addition, n-3 PUFAs are ligands of PPAR*γ*. Administration of n-3 PUFAs may enhance PPAR*γ* DNA-binding activity and consequently reduce downstream production of proinflammatory mediators [31]. With regard to polarization switch of macrophages, the lower M1/M2 ratio observed in the FO group after reperfusion may be explained by the property of n-3 PUFAs to modulate the Th1/Th2 balance towards the Th2 by suppression of Th1 response [32]. The designation of M1 and M2 macrophages was originally based on their ability to promote Th1 and Th2 responses, respectively; alternatively, Th1 and Th2 cytokine responses can also regulate M1/M2 polarization [13]. The process of muscle regeneration after injury involves an initial phase of necrotic debris removal at the site of injury by recruited “inflammatory” macrophages followed by a phase of fiber growth promotion which is marked by the switching of the M1 to the M2 macrophages [14]. It is plausible that the “elevated M2 phenomenon” observed in FO-pretreated animals can also be associated with improved tissue repair or resolution of injured muscles after IR injury.

In conclusion, this study demonstrated that limb ischemia resulted in the upregulation of proinflammatory cytokines and chemokines in the muscle, blood, and lungs 24 h after reperfusion. These proinflammatory mediators may orchestrate the subsequent migration and sequestration of neutrophils and monocytes/macrophages to the site of IR injury leading ultimately to local skeletal muscle and remote lung tissue damage. FO pretreatment modulated the increase of several proinflammatory cytokines and chemokines locally and systemically. Also, lowered M1/M2 ratio in the damaged muscle and higher Ly6C^−^ in blood were observed in response to FO pretreatment. These results suggest that FO pretreatment may have a protective role in limb IR injury not only by modulating the expression of proinflammatory cytokines and chemokines but also by regulating the polarization of macrophages.

## Figures and Tables

**Table 1:** Primer sequences used in the quantitative real-time reverse-transcription polymerase chain reaction assays.

Gene name	5′-3′ primer sequence
KC	F: CTTGAAGGTGTTGCCCTCAG
	R: TGGGGACACCTTTTAGCATC
MCP-1	F: ACTGAAGCCAGCTCTCTCTTCCTC
	R: TTCCTTCTTGGGGTCAGCACAGAC
TNF-*α*	F: CCCTCACACTCAGATCATCTTCT
	R: GCTACGACGTGGGCTACAG
IL-6	F: GGGACTGATGCTGGTGACAA
	R: ACAGGTCTGTTGGGAGTGGT
Hsp72	F: GCTGGCTAGGAGACAGATATGTGGC
	R: AAAGCCCACGTGCAATACACAAAGT
PPAR-*γ*	F: GCCCTTTGGTGACTTTATGG
	R: CAGCAGGTTGTCTTGGATGT
GAPDH	F: GAAGGTCGGTGTGAACGGAT
	R: AATCTCCACTTTGCCACTGC

KC, keratinocyte-derived chemokine; MCP-1, monocyte chemotactic protein-1; TNF-*α*, tumor necrosis factor-*α*; IL-6, interleukin-6; Hsp72, heat shock protein 72; PPAR-*γ*, peroxisome proliferator-activated receptor-*γ*; GAPDH, glyceraldehyde 3-phosphate dehydrogenase.

**Table 2:** Plasma concentrations of the cytokines.

	Sham	S0	F0	S24	F24
MCP-1 (pg/mL)	ND	5.04 ± 2.24	4.41 ± 2.07	16.4 ± 1.57^*∗*#^	7.69 ± 2.32^+^
MIP-2 (pg/mL)	0.38 ± 0.17	11.29 ± 4.29^*∗*^	7.03 ± 2.85	0.17 ± 0.11^#^	0.66 ± 0.31
IL-6 (pg/mL)	1.15 ± 0.57	6.35 ± 2.61	7.16 ± 2.63	39.5 ± 3.74^*∗*#^	23.37 ± 3.70^*∗*#^

ND: not detectable. Sham group, without IR procedure; S0 group, ischemic mice pretreated with saline injection sacrificed immediately after onset of reperfusion (time 0); F0 group, ischemic mice pretreated with FO emulsion sacrificed at time 0; S24, ischemic mice pretreated with saline injection sacrificed at 24 h after reperfusion (time 24 h); F24, ischemic mice pretreated with FO emulsion sacrificed at time 24 h. Data are presented as the mean ± SEM (*n* = 8). Differences among groups were analyzed by one-way ANOVA with Tukey's post hoc test. ^*∗*^Different from the sham group. ^+^Different from the saline (S) group at the same time point. ^#^Different from other time points in the same group (*p* < 0.05).
